# Frailty in end-stage renal disease: comparing patient, caregiver, and clinician perspectives

**DOI:** 10.1186/s12882-017-0558-x

**Published:** 2017-05-02

**Authors:** David A. Clark, Usman Khan, Bryce A. Kiberd, Colin C. Turner, Alison Dixon, David Landry, Heather C. Moffatt, Paige A. Moorhouse, Karthik K. Tennankore

**Affiliations:** 1 Division of Nephrology, Dalhousie University, 5070 Dickson Building, 5820 University Avenue, Halifax, B3H 2Y9 NS Canada; 2 Nova Scotia Health Authority, Halifax, NS Canada; 3 Division of Internal Medicine, Dalhousie University, Halifax, NS Canada; 4 Division of Geriatric Medicine, Dalhousie University, Halifax, NS Canada

**Keywords:** Frailty, Dialysis, Fried phenotype, Frailty index, Clinical frailty scale

## Abstract

**Background:**

Frailty is associated with poor outcomes for patients on dialysis and is traditionally measured using tools that assess physical impairment. Alternate measurement tools highlight cognitive and functional domains, requiring clinician, patient, and/or caregiver input. In this study, we compared frailty measures for incident dialysis patients that incorporate patient, clinician, and caregiver perspectives with an aim to contrast the measured prevalence of frailty using tools derived from different conceptual frameworks.

**Methods:**

A prospective cohort study of incident dialysis patients was conducted between February 2014 and June 2015. Frailty was assessed at dialysis onset using: 1) modified definition of Fried Phenotype (Dialysis Morbidity Mortality Study definition, DMMS); 2) Clinical Frailty Scale (CFS); 3) Frailty Assessment Care Planning Tool (provides CFS grading, FACT-CFS); and 4) Frailty Index (FI). Measures were compared via correlation and sensitivity/specificity analyses.

**Results:**

A total of 98 patients participated (mean age of 61 ± 14 years). Participants were primarily Caucasian (91%), male (58%), and the majority started on hemodialysis (83%). The median score for both the CFS and FACT-CFS was 4 (interquartile range of 3–5). The mean FI score was 0.31 (standard deviation ± 0.16). The DMMS identified 78% of patients as frail. The FACT-CFS demonstrated highest correlation (*r* = 0.71) with the FI, while the DMMS was most sensitive (97%, 100%) and a CFS ≥ 5 most specific (100%, 77%) at corresponding FI cutoff values (>0.21, >0.45).

**Conclusions:**

Frailty assessments of incident dialysis patients that include clinician, caregiver and patient perspectives have moderate to strong correlation with the FI. At specified FI cutoff values, the FACT-CFS and DMMS are highly sensitive measures of frailty. The CFS and FACT-CFS may represent viable alternative screening tools in dialysis patients.

**Electronic supplementary material:**

The online version of this article (doi:10.1186/s12882-017-0558-x) contains supplementary material, which is available to authorized users.

## Background

Frailty is typically described as a loss of functional, cognitive, and physiologic reserve leading to a vulnerable state [1, 2]. Studies examining frailty in dialysis populations have shown an increased risk for disability, hospitalization, and death in those identified as being frail [1, 3–7]. Identifying such individuals is of potential value in shared decision making and appropriate care.

In research settings, frailty is mostly operationalized by the Frailty Index or the Fried Phenotype [8], each of which is derived from different conceptual frameworks [1, 2, 9]. Fried [1] originally focused on function and fitness and used objective measurements (including grip strength testing, walking speed) as well as subjective self-report to determine frailty status. The Frailty Index classifies frailty status based on the proportion of a set of possible health deficits that are present in an individual patient (according to specific criteria) regardless of the domain or type of deficit involved [2]. While valuable, both methods can be cumbersome, lending difficulty to practical implementation in a clinical setting. The objective measurements originally put forth by Fried [1] are often cited as labor/resource intensive and have been thus substituted in part, using patient self-report [1, 3–6, 8, 10]. The Fried Frailty Phenotype is also difficult to apply when patients have depression, dementia, or Parkinson’s disease [2]. The Frailty Index, while highly precise on a population level, is difficult to apply to individual decision making discussions [11].

Less attention has been given to screening tools that rely on clinician impression or utilize collateral information to ascertain patient frailty status. In a prior study (at this institution) the Clinical Frailty Scale (CFS) was used to grade frailty severity in a cohort of dialysis patients and shown to have an association with mortality [3]. The CFS is derived from clinician impression (having had prior knowledge of patient comorbidities, cognitive health, and function) and includes cognitive deficits in the final score [12]. The Frailty Assessment for Care-planning Tool (FACT) method uses validated cognitive testing and caregiver input to provide information on patients’ cognitive state, functional status, mobility, and social interaction and can also be used to derive CFS scores [13].

Achieving a better understanding of how the assessment of frailty differs, using measures that are designed for patients, clinicians, caregivers, or a combination, may prove informative and eventually inform an optimal screening tool to be used in dialysis populations. It is possible that assessments derived from both clinician and caregiver input may better capture frailty. Therefore, in a cohort of incident dialysis patients, we aimed to contrast the measured prevalence of frailty using tools derived from different conceptual frameworks.

## Methods

### Study population

We conducted a prospective cohort study of all consecutive adult patients (age >18 years) who started chronic dialysis from February 15, 2014 to June 15, 2015, at a large tertiary care center. Patients on chronic dialysis were identified from a local electronic database as those who had end-stage renal disease requiring dialysis and would not be expected to recover kidney function. We included patients who initiated dialysis after failed kidney transplant. We excluded patients who only transiently received dialysis in our center (<1 week) with the intention and eventual transfer to another regional/out-of-province dialysis unit. All study participants provided written, informed consent.

### Measures of frailty

Frailty was evaluated using four different measures for all patients initiating dialysis during the noted timeframe.

#### Clinical Frailty Scale (CFS)

The modified version of the CFS was utilized in this study and is graded from 1 to 8 (1, very fit; 2, well without active disease; 3, well with treated co-morbid disease; 4, apparently vulnerable; 5, mildly frail; 6, moderately frail; 7, severely frail; 8, very severely frail) with a final category (9) to identify terminally ill patients who are not otherwise frail (Table 1). The CFS score was calculated by two different methods. A) Frailty level was ascertained on the basis of the clinician’s judgment and informed by the provided information for each level of frailty [12]. All ten nephrologists and three-nephrology nurse practitioners (at this institution) have been previously trained to determine the CFS using this methodology [3]. When dialysis initiation was scheduled in the out-patient clinic, a copy of the CFS, including the descriptors of each level, was provided to the primary nephrology practitioner, who prospectively evaluated CFS scores for each patient in the week preceding or day of dialysis. Among those who initiated chronic dialysis as an inpatient, the provider with the most clinical knowledge of the patient evaluated CFS scores prior to dialysis initiation. Among scheduled dialysis starts, this was the patient’s primary nephrologist or nephrology nurse practitioner. Among inpatient starts without prior follow-up, this was the attending/consulting nephrologist. B) The Frailty Assessment for Care-Planning Tool (FACT) utilizes collateral history in addition to patient response regarding mobility, function and social circumstances, as well as validated objective cognitive testing (Mini-Cog) [14], and memory axis of the Brief Cognitive Rating Scale [15] (Additional file 1). A graded score of 1 to 8, as per the modified version of the CFS [12], is assigned to each of these four domains, with the highest score in any given category determining the overall CFS score (FACT-CFS). A study investigator (UK) was trained to administer the FACT tool for all patients enrolled in this study. The FACT was administered within two weeks of dialysis initiation, at mid-session of a treatment, and not during the initial dialysis run, to minimize transient fluctuations in physical or cognitive function. If the patient was considered clinically unstable, the assessment was deferred to the next dialysis session. Among home hemodialysis and peritoneal dialysis patients, assessments were completed during the first week of dialysis training.

**Table 1:** The modified Canadian society of health and aging clinical frailty scale

CFS Score	Interpretation
1	Very fit: robust, active, energetic, well motivated, and fit; fittest in their age group
2	Well: without active disease but not as fit as those in category 1
3	Well: with treated comorbid disease
4	Apparently vulnerable: not dependent but has symptoms from comorbid disease (such as being slowed up)
5	Mildly frail: limited dependence on others for Instrumental activities of daily living
6	Moderately frail: help is needed for instrumental activities of daily living and activities of daily living
7	Severely frail: Completely dependent for personal care, from whatever cause (physical or cognitive). Even so, they seem stable and not at high risk of dying (within ~ 6 months).
8	Very severely frail: Completely dependent, approaching the end of life. Typically, they could not recover even from a minor illness.
9	Terminally Ill: Approaching the end of life. This category applies to people with a life expectancy <6 months, who are not otherwise evidently frail.

*CFS* Clinical frailty scale

#### Modified version of fried frailty phenotype

Frailty was assessed as per the methodology described by Johansen and colleagues in their analysis of the United States Renal Data System, Dialysis Morbidity and Mortality Study (DMMS) Wave II Cohort (DMMS-Frail, Table 2). Specifically, a score of <75 on the physical function scale of the Short Form-36 was used as a marker of weakness and slowness, a score of <55 on the vitality scale of the Short Form-36 was used to define poor endurance or exhaustion, and patients who reported that they “almost never” or “never” exercised were classified as inactive. Patients were classified as “undernourished” or not by the study investigator, using information available in the medical chart within 30 days before study entry and clinical examination at the time of frailty assessments. Using these criteria, a total of five points was possible, with two points for a low physical function score and one point for each of the other criteria. Patients scoring ≥3 were defined as frail [5]. DMMS-Frail assessments were carried out simultaneously with the FACT-CFS by the study investigator (UK), and frailty scores were calculated by a separate study investigator who was blinded to the results of the FACT-CFS or CFS (KT) using designated software upon completion.

**Table 2:** DMMS^a^ Wave II definition of Frailty, component criteria

Criteria	Score
Short Form-36 Vitality Score <55.	1 point
Undernourished or cachectic as assessed by data abstractor.	1 point
Answer to following question: How often do you exercise? Almost never or never?	1 point
Short Form-36 Physical Function Score <75	2 points

Score ≥3 defined as frail

^a^Dialysis Morbidity and Mortality Study

#### Frailty index

A Frailty Index was generated using standard methodology [16]. Only binary variables were utilized, and coded using the convention that ‘0’ indicated the absence of the deficit, and ‘1’ the presence of a deficit. A total of 32 variables were derived from source data including baseline characteristics and frailty measure questionnaire responses (Additional file 2). The Frailty Index served as the reference standard for other measures in this study as previously performed in studies involving geriatric and end stage renal disease populations [12, 16, 17].

### Baseline characteristics

Characteristics, including age, sex, race, height, weight, comorbidities (diabetes, hypertension, coronary artery disease, peripheral vascular disease, cerebral vascular disease, chronic lung disease, connective tissue disease, malignancy), cause of end-stage renal disease (ESRD), type of dialysis (peritoneal, home hemodialysis, hemodialysis with central venous catheter, or hemodialysis with arteriovenous fistula or graft) and laboratory data (hemoglobin, phosphorous, calcium, albumin, creatinine) were collected at the start of dialysis by the patient’s primary nephrologist on the basis of clinical knowledge and documentation in electronic records. Estimated glomerular Filtration Rate (eGFR) was calculated using the four-variable Modified Diet in Renal Disease (MDRD) equation at the start of dialysis on the basis of serum creatinine values on the day of dialysis initiation. If laboratory results were not available on the day of dialysis, the most recent values within the preceding month were used. Missing data values were addressed by re-examination of electronic records (DC).

### Statistical analyses

Descriptive statistics were reported as counts and percentages for categorical variables, means ± standard deviation for normally distributed continuous variables, and medians and interquartile ranges for non-normally distributed continuous variables. The CFS and FACT-CFS scores were treated as continuous variables with a fixed interval between each scale score [3]. Normality was assessed using the Shapiro Wilk test. Pearson correlation was used to compare the Frailty Index and both derivations of the CFS. Comparison with the DMMS-Frail assessment was performed using point bi-serial correlation. In pre-specified sensitivity analyses, we evaluated sensitivity/specificity of the CFS, FACT-CFS, and DMMS-Frail compared with the Frailty Index as the reference standard. The Frailty Index was dichotomized at previously studied cut-off scores >0.21 (frail) and >0.45 (most frail) [18]. The CFS and FACT-CFS were evaluated at threshold scores of 4 (apparently vulnerable) and 5 (mildly frail).

Statistical analyses were performed using Stata IC, version 12 (StatCorp, College Station, TX) A two-sided p value of <0.05 was the threshold for statistical significance. Approval for this study was received from the Nova Scotia Health Authority Research Ethics Board.

## Results

### Study population

**Fig. 1:**
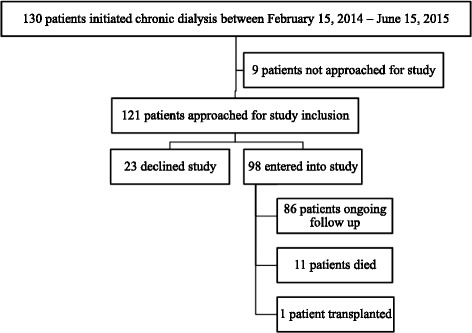
Cohort Selection

During the study period, 130 patients started chronic dialysis, of whom 98 (75%) consented to participate (Fig. 1). Of these individuals, 11 died and one received a kidney transplant. Nine patients only transiently received dialysis in our center (<1 week) and were not approached for study inclusion. Baseline characteristics of participants are displayed in Table 3.

**Table 3:** Baseline Characteristics of patients^a^, *n* = 98

Demographics	
Age (mean years ± SD)	61 + 14
Male, n (%)	57 (58)
Caucasian, n (%)	89 (91)
Cause of End Stage Renal Disease, n (%)	
Diabetes	27 (28)
Other	29 (30)
Ischemic/ Hypertension	9 (9)
Unknown	7 (7)
Glomerulonephritis	19 (19)
Polycystic kidney disease	7 (7)
Comorbid Conditions, n (%)	
Diabetes	46 (47)
Coronary artery disease	27 (28)
Congestive heart failure	20 (20)
Peripheral vascular disease	10 (10)
Chronic lung disease	13 (13)
Cerebrovascular disease	6 (6)
Connective tissue disease	5 (5)
Neoplasia	12 (12)
Peptic ulcer disease	9 (9)
Laboratory	
MDRD GFR^b^ [median mL/min/1.73 m^2^ (Q1-Q3)]	8 (6–11)
Albumin [median g/L (Q1-Q3)]	32 (27–34)
Creatinine [median umol/L (Q1-Q3)]	567 (447–764)
Phosphate [median mmol/L (Q1-Q3)]	1.84 (1.56–2.14)
Hemoglobin (mean g/L ± SD)	89 + 13
Dialysis Modality, n (%)	
In center/satellite hemodialysis	80 (82)
Home hemodialysis	3 (3)
Peritoneal dialysis	15 (15)
Hemodialysis Access, n (%)^c^	
Hemodialysis with central venous catheter	61 (73)
Hemodialysis with arteriovenous fistula	22 (27)
Other	
BMI^d^ [median kg/m^2^ (Q1-Q3)]	28 (25–32)
Initial dialysis as inpatient, n (%)^e^	33 (34)
Late referral, n (%)^f^	14 (14)

^a^No patients documented as having a diagnosis of dementia

^b^Modification of diet in renal disease glomerular filtration rate

^c^ *N* = 83

^d^Body mass index

^e^All peritoneal and home hemodialysis patients initiated as outpatients

^f^Defined as >3 months before assessed by a nephrologist

### Frailty measures

**Fig. 2:**
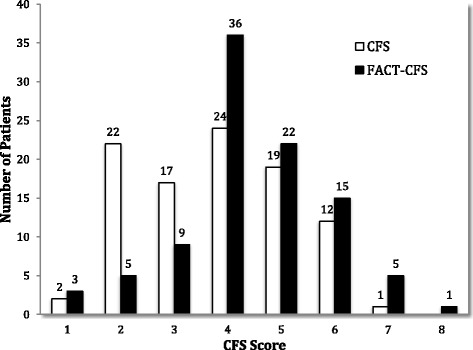
Distribution of CFS and FACT-CFS scores. CFS, Clinical Frailty Scale. FACT, Frailty Assessment for Care-Planning Tool

Median scores for the CFS, FACT-CFS, and FACT-CFS subcategories are listed in Table 4. CFS and FACT-CFS were normally distributed (*P* = 0.19 and 0.70 respectively). The distribution of CFS and FACT-CFS scores are displayed in Fig. 2. The FACT-CFS identified 44% of patients as being mildly to severely frail (scores of 5–7) versus 33% of patients using the CFS. One patient was classified as being very severely frail by the FACT-CFS (score = 8). More patients were apparently vulnerable (score = 4) using the FACT-CFS (38%) compared to the CFS (25%). The Short Form-36 and individual self-report component scores used in the DMMS Wave II frailty definition are reported in Table 5. Overall 78% of patients were classified as frail and 97% of individuals reported physical inactivity.

**Table 4:** Comparison of frailty scale measures

n	Frailty Measure [median score (Q1-Q3)]	
97	CFS	4 (3–5)
96	FACT-CFS	4 (4–5)
	Mobility^a^	4 (3–5)
	Function^a^	4 (3–5)
	Cognition^a^	1 (1–3)
	Social^a^	3 (1–4)

*CFS* Clinical frailty scale

*FACT* Frailty assessment for care-planning tool

^a^FACT Subcategories

**Table 5:** Results of the DMMS^a^ Wave II definition of frailty including Short Form-36 and individual component scores *n* = 96

Short Form-36 Scale Scores [median (Q1-Q3)]	
Physical Functioning	45 (25–75)
Role Limitations due to Physical Problems	31 (13–56)
Bodily Pain	52 (31–84)
General Health	35 (24–51)
Vitality	34 (19–56)
Social Functioning	56 (38–81)
Role Limitations due to Emotional Problems^b^	75 (42–100)
General Mental Health	72 (60–85)
Physical Component Summary^b^	35 (29–43)
Mental Component Summary^b^	48 (38–54)
Poor Endurance/Exhaustion, n (%)	69 (72)
Unintentional Weight Loss, n (%)	10 (10)
Physical Inactivity, n (%)	93 (97)
Slowness/Weakness, n (%)	75 (78)
DMMS Wave II Definition of Frailty, n (%)	75 (78)

^a^Dialysis Morbidity and Mortality Study

^b^ *n* = 95

The mean Frailty Index value for this study population was 0.31 ± 0.16 (*n* = 98). The Frailty Index followed a normal distribution (*P* = 0.39). Correlation between each frailty instrument is depicted in Table 6. Overall, the FACT-CFS demonstrated the highest correlation with the Frailty Index (*r* = 0.71). The CFS, DMMS-Frail, and FACT-CFS function and mobility subcategories had moderate correlation with the Frailty Index (Table 6).

**Table 6:** Correlations between the assessment results of each frailty measure

	Frailty Index	CFS	FACT-CFS	DMMS-Frail	FACT-CFS Function^a^	FACT-CFS Mobility^a^	FACT-CFS Cognition^a^	FACT-CFS Social^a^
Frailty Index	1	0.57	0.71	0.64	0.68	0.67	0.39	0.35
CFS	0.57	1	0.45	0.43	0.33	0.47	0.26	0.18
FACT-CFS	0.71	0.45	1	0.47	0.75	0.78	0.43	0.53
DMMS-Frail	0.64	0.43	0.47	1	0.44	0.44	0.27	0.22
FACT-CFS Function^a^	0.68	0.33	0.75	0.44	1	0.61	0.34	0.37
FACT-CFS Mobility^a^	0.67	0.47	0.78	0.44	0.61	1	0.18	0.35
FACT-CFS Cognition^a^	0.39	0.26	0.43	0.27	0.34	0.18	1	0.11
FACT-CFS Social^a^	0.35	0.18	0.53	0.22	0.37	0.35	0.11	1

*CFS* Clinical frailty scale, *FACT* Frailty assessment for care-planning tool, *DMMS* Dialysis morbidity and mortality study

^a^FACT Subcategories

### Sensitivity analyses

Sensitivity, specificity, and positive and negative predictive values for the CFS, DMMS-Frail, and FACT-CFS as compared with the Frailty Index are listed in Table 7. The DMMS-Frail was most sensitive at both chosen levels of frailty (Table 7).

**Table 7:** Comparison of screening performance measures^a^ of frailty assessment instruments

	FI >0.21	FI >0.45
CFS ≥4		
Sensitivity	71 (58–81)	90 (68–99)
Specificity	72 (53–87)	51 (39–62)
PPV	86 (77–92)	31 (26–37)
NPV	51 (41–62)	95 (84–99)
CFS ≥5		
Sensitivity	47 (35–60)	70 (46–88)
Specificity	100 (88–100)	77 (66–86)
PPV	100 (81–100)	43 (31–55)
NPV	45 (39–50)	91 (84–95)
FACT-CFS ≥4		
Sensitivity	94 (86–98)	95 (75–100)
Specificity	48 (29–68)	21 (13–32)
PPV	81 (75–86)	23 (21–26)
NPV	78 (56–91)	94 (70–99)
FACT-CFS ≥5		
Sensitivity	62 (50–74)	85 (62–97)
Specificity	100 (87–100)	66 (54–76)
PPV	100 (84–100)	38 (30–47)
NPV	53 (45–60)	95 (86–98)
DMMS-Frail		
Sensitivity	97 (90–100)	100 (83–100)
Specificity	70 (50–86)	28 (18–39)
PPV	88 (81–93)	26 (23–28)
NPV	91 (72–98)	100 (75–100)

*CFS* Clinical Frailty Scale, *FACT* Frailty Assessment for Care-Planning Tool, *DMMS* Dialysis Morbidity and Mortality Study, *FI* Frailty Index, *PPV* Positive Predictive Value, *NPV* Negative Predictive Value

^a^Values reported as percentages

## Discussion

This study explores differences in frailty perception in the dialysis population, by comparing screening instruments designed for patients, clinicians, and/or caregivers, and that differ by conceptual framework. Of the screening instruments studied, all were moderately correlated with the Frailty Index, but the FACT tool, which incorporates collateral perspective as well as objective cognitive testing, had the highest correlation. In contrast, the DMMS definition of frailty, a modified version of the Fried Frailty Phenotype, had the highest sensitivity for screening for frailty in this study population.

In this study, a constructed Frailty Index identified approximately 70% of patients as frail (index score >0.21). In contrast to the characteristic gamma distribution displayed in prior Frailty Index analyses of community dwelling older adults [2, 16], we observed a normal distribution in our cohort, potentially reflecting the higher disease burden seen in the dialysis population. Conversely, a characteristic age-independent frailty limit was still evident, demonstrated by a maximum deficit accumulation of <0.7.

Both we, and other investigators report a high prevalence of frailty when using a modified definition of the Fried Phenotype [5, 7]. In this study, a modified definition was moderately correlated with the Frailty Index and was calculated to be a highly sensitive screening tool. However, in clinical application, the modified Fried Phenotype definition may lack discriminatory power, as an over-inclusive measure. Other studies have shown that when compared to the original criteria set forth by Fried [1], a modified definition which substitutes physical performance measures with patient self-report classifies more patients as frail [19, 20]. In addition, the modified definition of the Fried Phenotype does not grade frailty severity at the individual level, or take into account cognitive health. Nevertheless, the presence of frailty as defined by the DMMS-Frail was 97% (90–100) and 100% (83–100) sensitive using frailty index cut-offs of >0.21 and >0.45.

Using the CFS, the prevalence of frailty in this study was 33%. If subjects classified as apparently vulnerable (CFS score = 4) were included, 58% were frail. These findings are comparable with a prior study performed at this center [3], which observed 26% of 390 chronic dialysis patients classified as frail using the CFS, and 53% if apparently vulnerable subjects included. In this study, the CFS was moderately correlated with the Frailty Index (*r* = 0.57). In comparison, Rockwood *et al*.[12] observed a high correlation (*r* = 0.8) when analyzing a large cohort of community dwelling older adults. Given that the CFS is derived from an overall assessment based on clinician impression, it is plausible that nephrology-trained professionals may under diagnose frailty relative to geriatricians or clinicians with additional geriatric training. This is denoted by the observation that no patients included in this study were identified as having dementia at study inclusion. In contrast, the FACT tool led to derived CFS scores with higher correlation to the Frailty Index (*r* = 0.71) and improved sensitivity for frailty detection (Table 7).

In this study, we identify potential alternative frailty measures for both screening and/or assessment purposes in dialysis patients. As outlined by Rockwood *et al.*[21], both types of frailty measures (for screening and for assessment) are needed, depending on whether the goal is to identify individuals/populations at risk, or to grade the severity of frailty for potential targeted interventions when the degree of risk varies. To that end, the Clinical Frailty Scale and Frailty Assessment for Care-Planning Tool are not resource intensive, serving both purposes and may serve as alternative screening tools. Larger prospective and longitudinal studies are needed to assess the performance of these different frailty instruments, also necessitating further study of the natural history of frail dialysis patients, and effect(s), if any, of potential targeted interventions [3–5, 9, 22].

The estimated prevalence of frailty in this study ranged between 33% and 78%. A recent study assessing frailty as measured by six different screening tools in a prospective cohort of Taiwanese dialysis patients also reported a comparable, wide prevalence range of 30% to 82%[10]. It is likely that both the heterogeneity of the frailty syndrome, and thus assortment of developed assessment tools, are at least partially responsible for the observed variation. Furthermore, the heterogenous nature of our study population with respect to function, having included both home dialysis patients as well as patients initiated on hemodialysis either on an inpatient or outpatient basis, may have also contributed to the observed variation in measured frailty prevalence. Ultimately, it is still unclear which instrument(s) is best for assessing and/or screening for frailty in dialysis cohorts. Given that the evaluation of instrument performance, including those utilized in this study, is hampered by the lack of a gold standard in the scientific literature, importance will be in future efforts to formulate a consensus definition of frailty.

This study has several strengths. We prospectively evaluated frailty. A total of four frailty screening instruments spanning alternate operational frameworks and capturing patient, caregiver, and/or health care provider perspective(s) were included for comparison. The prospective acquisition of CFS scores improved the accuracy of data collection by limiting the possibility of misclassification of patients. We also had a reasonable representation of patients with completed CFS scores.

Despite these strengths, there are limitations. This is a single center study. Our findings would need to be assessed in larger longitudinal studies examining also for relevant associated outcomes (i.e. use of health care resources, morbidity, mortality), followed by potential therapeutic intervention(s). In this study, frailty was assessed relatively early after dialysis initiation. Therefore, despite outlined efforts to minimize effects of transient fluctuations in physical or cognitive function on frailty assessment, it is possible that the impact of common end stage renal disease comorbidities may have been transiently heightened as suggested by the lower mean hemoglobin observed in our cohort. Furthermore we, at our center, have analyzed the CFS in two dialysis cohorts, both in the present study and one previous [3], however the CFS should be validated in dialysis cohorts at other centers. It is plausible that dialysis programs with less familiarity with the CFS may observe different performance measures. Finally, it is likely that many patients who were severely frail may not have been considered for dialysis and thus not included in this analysis. However, given that 22% of patients in this study had CFS scores ≥6 (moderately frail) as determined by the FACT-CFS, it is unlikely that frail status precluded dialysis initiation.

## Conclusions

Frailty assessments of incident chronic dialysis patients that utilize clinician, caregiver and patient perspectives are moderately to strongly correlated with a Frailty Index. The Frailty Assessment for Care-Planning tool, as well as the DMMS Wave II study definition of frailty, are highly sensitive for diagnosing frailty using different Frailty Index cut-offs. The Clinical Frailty Scale and Frailty Assessment for Care-Planning Tool are not resource intensive and may represent viable alternative screening tools, however larger prospective and longitudinal studies are ultimately needed to assess their performance in the dialysis population.

## Additional files

Additional file 1: Frailty assessment for care planning tool (FACT). Copy of the Frailty Assessment for Care-Planning Tool (FACT) which utilizes collateral history in addition to patient response regarding mobility, function and social circumstances, as well as objective cognitive testing, and memory axis of the Brief Cognitive Rating Scale. (DOCX 489 kb)

Additional file 2: Binary variables utilized to construct Frailty Index. Listing of 32 binary variables utilized to construct the Frailty Index. (DOCX 21 kb)

## Abbreviations

CFS: Clinical frailty scale
DMMS: Dialysis morbidity mortality study definition
eGFR: Estimated glomerular filtration rate
ESRD: End-stage renal disease
FACT: Frailty assessment care planning tool
FI: Frailty index
MDRD: Modified diet in renal disease
