# Supplementary figures and images for: Frailty in end-stage renal disease: comparing patient, caregiver, and clinician perspectives
Source: BMC Nephrol. 2017 May 2;18:148. doi: 10.1186/s12882-017-0558-x (PMC5412047; doi:10.1186/s12882-017-0558-x)

**Additional file 1. Frailty assessment for care planning tool (FACT)**

**
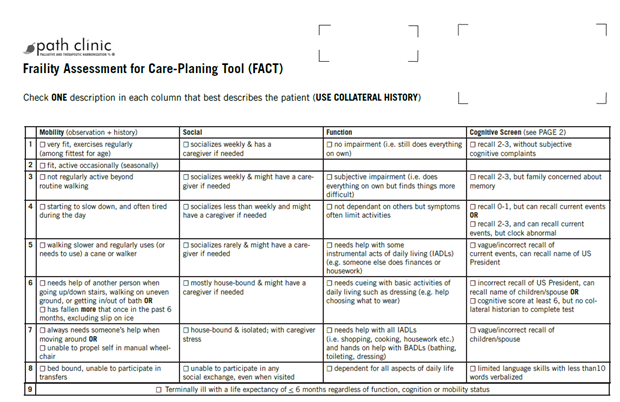
**


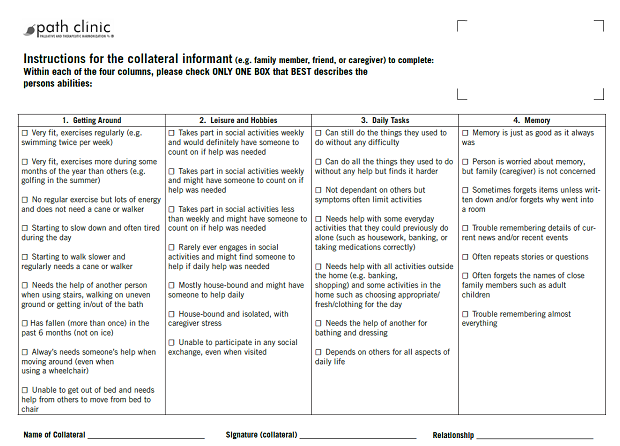


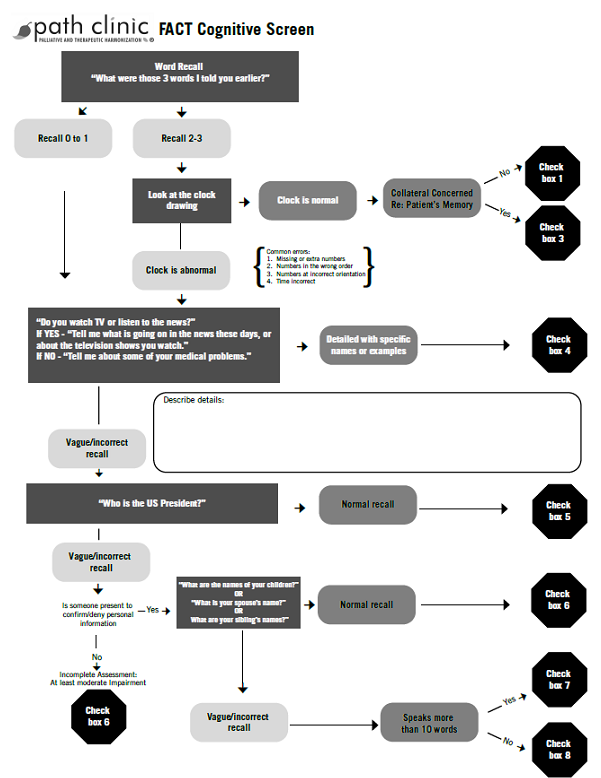

Supplement: Supplementary file 1 — Frailty assessment for care planning tool (FACT). Copy of the Frailty Assessment for Care-Planning Tool (FACT) which utilizes collateral history in addition to patient response regarding mobility, function and social circumstances, as well as objective cognitive testing, and memory axis of the Brief Cognitive Rating Scale. (DOCX 489 kb) [file 12882_2017_558_MOESM1_ESM.docx]
