# Supplementary material for: Frailty in end-stage renal disease: comparing patient, caregiver, and clinician perspectives
Source: BMC Nephrol. 2017 May 2;18:148. doi: 10.1186/s12882-017-0558-x (PMC5412047; doi:10.1186/s12882-017-0558-x)
Supplement: Supplementary file 2 — Binary variables utilized to construct Frailty Index. Listing of 32 binary variables utilized to construct the Frailty Index. (DOCX 21 kb) [file 12882_2017_558_MOESM2_ESM.docx]

**Additional file 2. Binary variables utilized to construct Frailty Index**

| **Comorbidities/clinical data** |
| --- |
| Coronary artery disease |
| Congestive heart failure |
| Peripheral vascular disease |
| Cerebrovascular disease |
| COPD |
| Connective tissue disease |
| Diabetes |
| Peripheral vascular disease |
| Liver disease |
| Cancer |
| Albumin <32 g/L |
| Walking slower and uses (or should use) cane/walker; or worse |
| Socializes rarely; or worse |
| Needs help with some instrumental activities of daily living; or worse |
| Vague/incorrect recall of current events; or worse |
| Abnormal clock drawing |
| Unintentional weight loss |
| Fair/poor self rating of health |
| Health is worse in last year |
| Limited to perform moderate activities |
| Limited lifting/carrying groceries |
| Walking one hundred meters |
| Limited climbing one flight of stairs |
| Limited bending, kneeling, or stooping |
| Limited bathing or dressing |
| Cut down amount of time at work/on activities most/all of the time due to  physical health |
| Cut down amount of time at work or other activities most/all of the time due to  emotional problems |
| Physical or emotional problems interfere with social activities quite a  bit/extremely |
| Pain interfered with normal work (in/out of home) quite a bit/extremely |
| Feel very nervous most/all of the time |
| Feel downhearted and depressed most/all of the time  Feel worn out most/all of the time |
